# Successful surgical drainage with intraoperative ultrasonography for amebic liver abscess refractory to metronidazole and percutaneous drainage: a case report

**DOI:** 10.1186/s12893-020-00776-x

**Published:** 2020-05-24

**Authors:** Keita Kouzu, Takahiro Einama, Makoto Nishikawa, Makiko Fukumura, Hiromi Nagata, Toshimitsu Iwasaki, Yoichi Miyata, Yasuhiro Obuchi, Kazuo Hase, Hideki Ueno, Yoji Kishi, Junji Yamamoto

**Affiliations:** 1 Department of Surgery, National Defense Medical College, 3-2 Namiki, Tokorozawa, Saitama, 359-8513 Japan; 2 Department of General Medicine, National Defense Medical College, 3-2 Namiki, Tokorozawa, Saitama, 359-8513 Japan; 3 Department of Surgery, New-Tokyo Hospital, 1271, Wanagaya, Matsudo, Chiba, 270-2232 Japan

**Keywords:** Amebic liver abscess, Surgical drainage, Intraoperative ultrasonography, Decompression, *Entamoeba histolytica*

## Abstract

**Background:**

Metronidazole (MNZ) has been clearly established as a medication for amebic liver abscess. In uncomplicated cases, surgical drainage should be avoided. We report a case of amebic liver abscess refractory to MNZ that was successfully treated using preoperative computed tomography (CT) and percutaneous and surgical drainage with intraoperative ultrasonography (IOUS).

**Case presentation:**

A 53-year-old man with high-grade fever was diagnosed with a cystic lesion on his right hepatic lobe using CT. Percutaneous drainage was performed, and antibacterial drugs were administered. However, the infection and condition of the patient worsened. *Entamoeba histolytica* was detected from pus within the mediastinal cavity. Hence, the patient was diagnosed with amebic liver abscess. After the diagnosis was established, we administered MNZ for 10 days. Despite this, the patient’s physical condition did not improve. Blood tests suggested impending disseminated intravascular coagulation (DIC). We performed surgical intervention to drain the amebic liver abscess refractory to conservative treatment. During surgery, imaging information from preoperative CT and IOUS enabled us to recognize the anatomical structures and determine the incision lines of the hepatic capsule and hepatic tissue. The patient’s DIC immediately regressed after surgery. Unfortunately, malnutrition and disuse syndrome contributed to the patient’s long recovery period. He was discharged 137 days post-surgery.

**Conclusions:**

We reported a case of amebic liver abscess refractory to conservative treatment. Surgical drainage with preoperative CT and IOUS allowed us to safely and effectively perform complex abscess decompression.

## Background

Amebiasis is the parasitic disease of *Entamoeba histolytica.* It is the major cause of death from parasitic disease infection [1]. Amebic liver abscess is the most common extraintestinal manifestation of amebic infection. Metronidazole (MNZ) is a clearly established, safe, and effective medication. Percutaneous drainage is usually not indicated, and surgical drainage should be avoided for uncomplicated amebic liver abscesses [2, 3]. We report a case of amebic liver abscess refractory to MNZ. We successfully treated the patient using percutaneous and surgical drainage with preoperative computed tomography (CT) and intraoperative ultrasonography (IOUS).

## Case presentation

A 53-year-old man was admitted to a nearby hospital owing to high-grade fever. CT revealed a low-density mass in his right hepatic lobe. Consequently, he was diagnosed with a bacterial liver abscess. Percutaneous drainage of the hepatic abscess was performed, as well as treatment with antibacterial drugs. Despite this, the infection and condition of the patient worsened. He was admitted to our hospital for undergoing intensive treatment (Table 1).

**Table 1:** Blood tests when transferred to our hospital

Total bilirubin	0.59	mg/dL	White blood cell	17,600	/uL
AST	182	IU/L	Hemoglobin	15.0	g/dL
ALT	421	IU/L	Hematocrit	33.6	%
Total protein	5.2	g/dL	Platelets	72.0	× 10^4^/uL
Albumin	1.3	g/dL	PT-INR	2.0	
Blood sugar	120	mg/dL	APTT	40.0	sec (INR)
Total Cholesterol	33	mg/dL	FDP	20	ug/mL
Na	129	mmol/L	BUN	61	mg/dL
K	4.3	mmol/L	Creatinine	1.0	mg/dL
Cl	92	mmol/L	CRP	32.0	mg/dL

*AST* aspartate aminotransferase, *ALT* alanine aminotransferase, *BUN* blood urea nitrogen, *PT* prothrombin time, *APTT* activated partial thromboplastin time, *FDP* fibrin degradation product, *INR* international normalized ratio

**Fig. 1:**
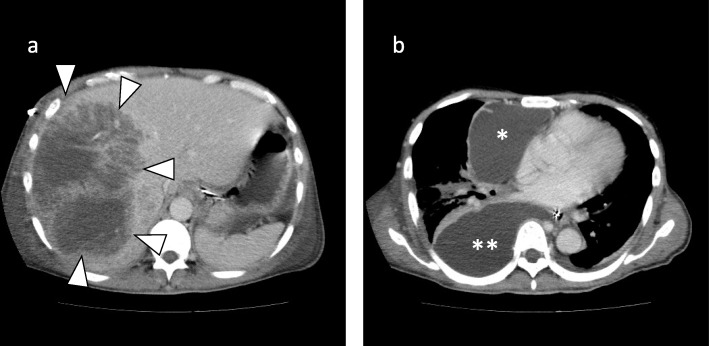
**a** CT showing a liver abscess in the right liver lobe partially containing necrotic solid tissue. **b** The liquid abscess cavities in the mediastinum (*) and right thoracic cavity (**). CT: Computed tomography

His vital signs included a temperature of 38.8 °C, blood pressure of 122/87 mmHg, heart rate of 120 beats per minutes, and disturbance of consciousness (Glasgow coma scale, 13). The patient had no pertinent medical history other than pneumothorax. He had no history of sexual activity nor travel. At the time of admission, no pathogen was identified from the pus of the liver abscess or the blood culture. CT demonstrated that the low-density, 15-cm mass in the right liver lobe was partially replaced by necrotic solid tissue. There was an emergence of new fluid lesions in the mediastinum and right thoracic cavity (Fig. 1). After admission to our hospital, additional percutaneous drainage of both fluid lesions was performed. *E. histolytica* was detected from the drained discharge of the mediastinal cavity. Thus, the patient was diagnosed with amebic liver abscess. We administered MNZ (1500 mg) orally for 10 days, but his physical condition did not improve. The patient presented with a sustained fever of 38.9 °C, blood pressure of 117/79 mmHg, heart rate of 117 beats per minutes, respiratory rate of 32 per minutes, and a disturbed consciousness (Glasgow coma scale, 13). The patient had abdominal distention and complained of widespread abdominal pain. Additionally, his laboratory blood tests revealed a high inflammatory reaction and a tendency towards disseminated intravascular coagulation (Table 2). CT showed no change in the size of the liver abscess (15 cm). The mediastinal abscess was already well drained. No additional drainage was performed during surgery (Fig. 4). Based on these results, we decided to perform surgical intervention to drain the amebic liver abscess.

**Table 2:** Blood tests just before emergency operation

Total bilirubin	1.03	mg/dL	White blood cell	24,700	/uL
AST	65	IU/L	Hemoglobin	7.1	g/dL
ALT	36	IU/L	Hematocrit	21.4	%
Total protein	5.5	g/dL	Platelets	28.8	×10^4^/uL
Albumin	1.4	g/dL	PT-INR	1.58	
Blood sugar	114	mg/dL	APTT	44.2	sec (INR)
Total Cholesterol	37	mg/dL	FDP	33	ug/mL
Na	137	mmol/L	BUN	14	mg/dL
K	3.4	mmol/L	Creatinine	0.39	mg/dL
Cl	102	mmol/L	CRP	9.9	mg/dL

*AST* aspartate aminotransferase, *ALT* alanine aminotransferase, *BUN* blood urea nitrogen, *PT* prothrombin time, *APTT* activated partial thromboplastin time, *FDP* fibrin degradation product, *INR* international normalized ratio

**Fig. 2:**
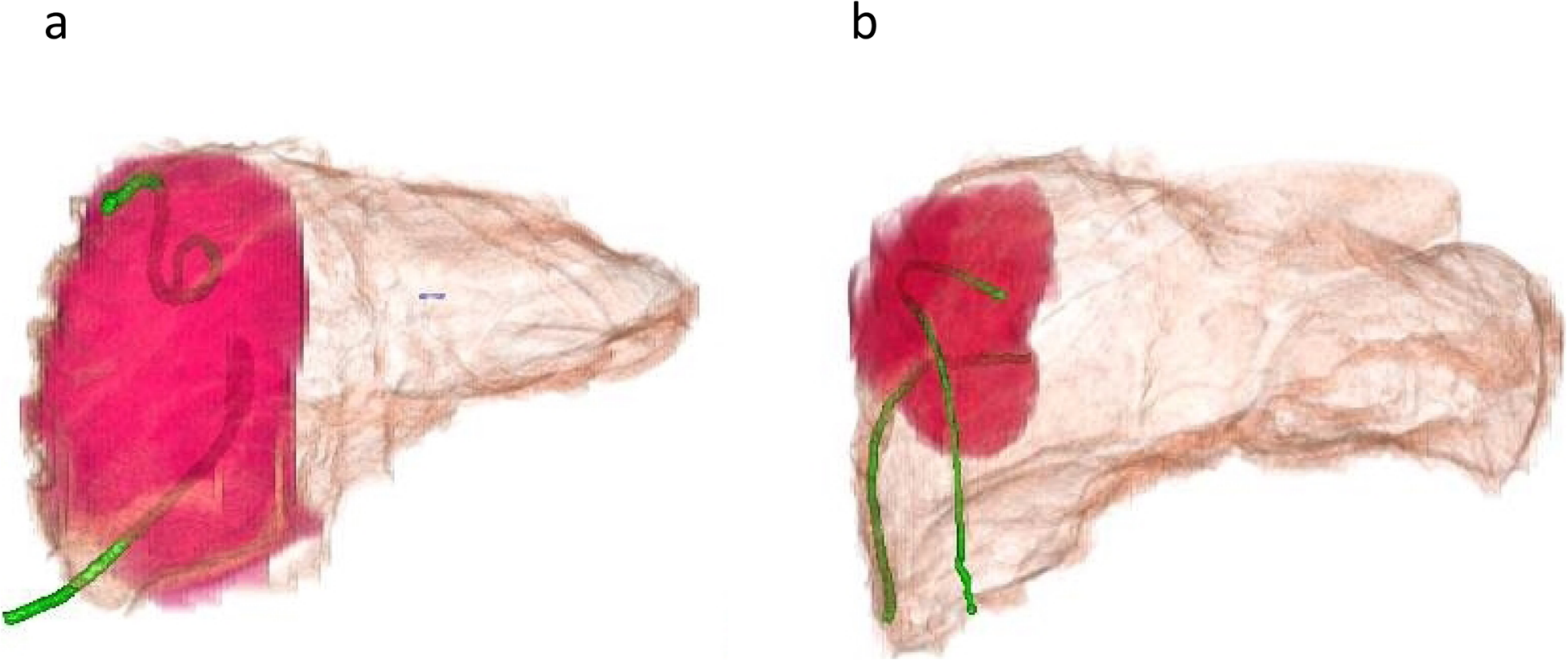
**a** Preoperative CT. **b** Postoperative CT. Red area: Abscess, Green line: (**a**) Catheter and (**b**) drain tube. CT: Computed tomography

**Fig. 3:**
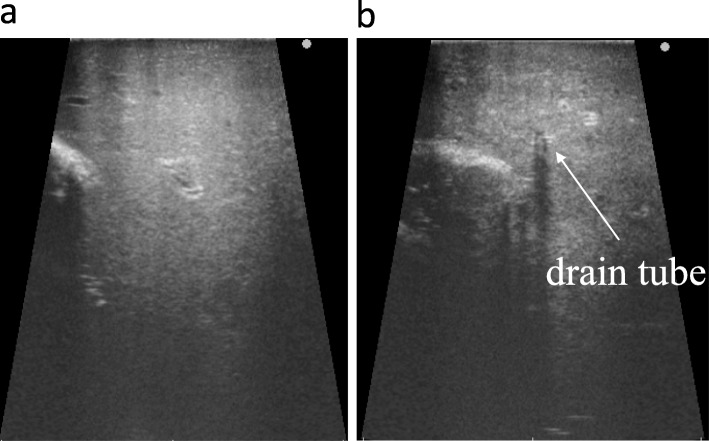
Photographs of intraoperative ultrasonography. **a** The normal liver, and **b** the necrotic liver and drain tube

**Fig. 4:**
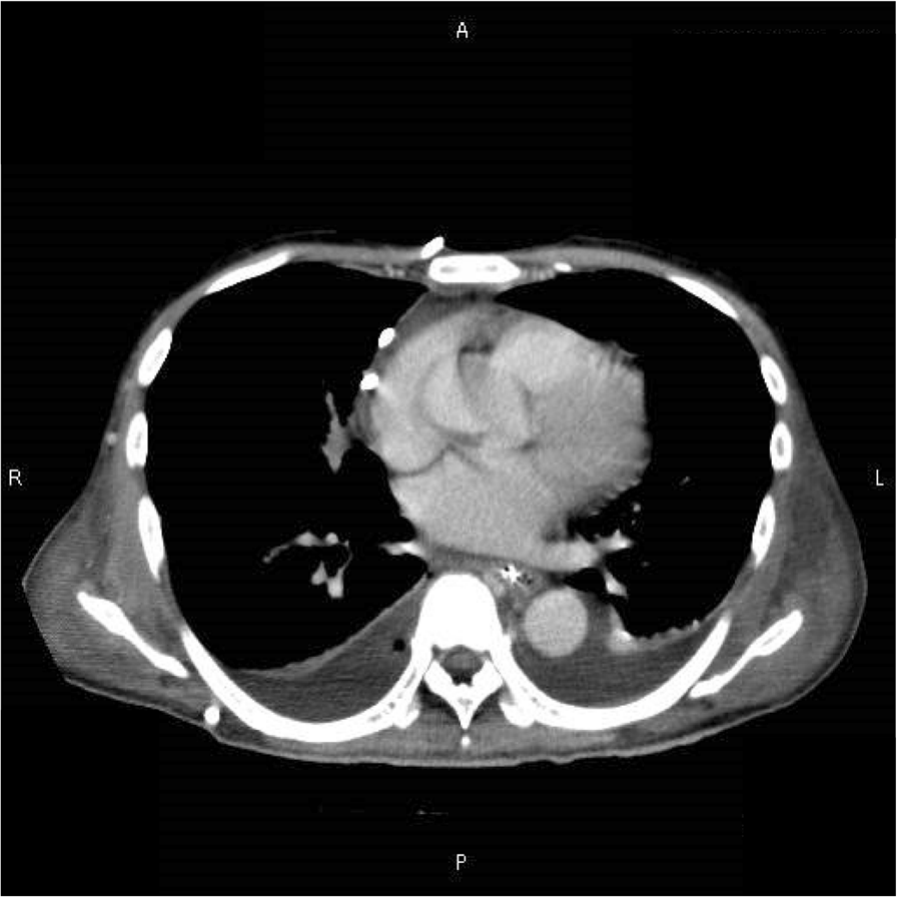
The mediastinal abscess was already well drained when surgery was performed

On laparotomy, there was a congested peritoneum due to acute secondary diffuse suppurative peritonitis not only around the liver, but also around the abdominal cavity. The small intestine was dilated because of inflammation. The coagulation abnormality induced easy bleeding, especially at the time of right liver mobilization. The boundary of the liver abscess could not be detected macroscopically since the visible change in the hepatic capsule was minimal. However, preoperative CT allowed us to estimate the range of the cavity (Fig. 2a). The aspiration drains became important guideposts to reference CT images. We also used IOUS to determine adequate resection areas of the hepatic capsule and hepatic tissue (Fig. 3). These modalities allowed us to perform a safe necrotic tissue resection without damage to the major vessels. The whitish and semi-solid necrotic tissue occupied most of the abscess cavity in the right liver lobe. This was considered the cause of inefficient drainage and was debrided. The pig tail catheters inserted in the abscess were replaced with thick drains. A jejunostomy was done using a needle catheter to create a route for nutrient administration (Fig. 2b). Because the patient had coagulopathy, stopping liver bleeding posed a difficult problem. Hence, resection or enucleation of the abscess area was not performed. The mediastinal abscess was already well-drained. No additional drainage was performed during surgery (Fig. 4). The operation time was 246 min, with intraoperative bleeding amounting to 2635 mL. The excised specimen pathologically showed purulent granulomas, although the presence of amoeba could not be confirmed. *E. histolytica* has not been detected again in any drain since the initial diagnosis.

**Fig. 5:**
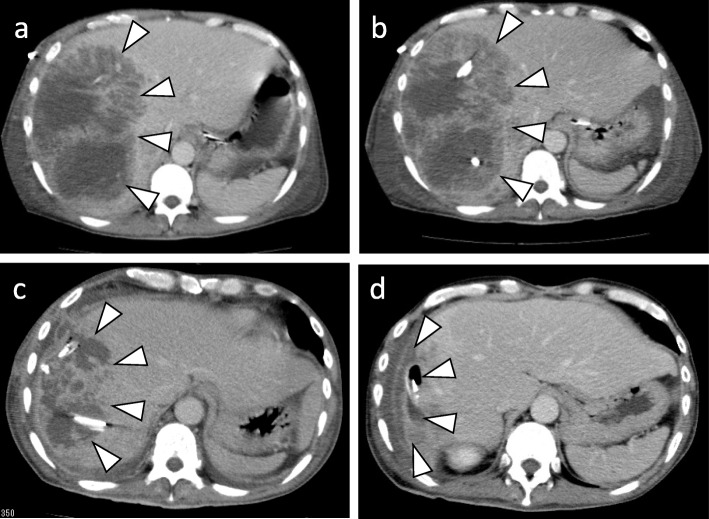
CT showing that the abscess reduced in size. **a** CT when the patient was admitted. **b** Preoperative CT. **c** Postoperative CT. **d** CT before the patient was discharged. CT: Computed tomography

The disseminated intravascular coagulation immediately subsided after surgery. Owing to a high fever, the patient was placed on prolonged bedrest. He eventually became malnourished and suffered from disuse syndrome. After long-term physical therapy, the patient was discharged 137 days after surgery. In the CT prior to his discharge, the abscess had almost disappeared (Fig. 5). The patient had no recurrence of liver abscess 1 year after surgery.

## Discussion and conclusions

We encountered a case of amebic liver abscess effectively treated with surgical drainage using preoperative CT and IOUS. Although the large abscess showed resistance to MNZ treatment, the patient was saved through careful planning and correct device usage during the operation. To our knowledge, this is the first report to demonstrate the effectiveness of surgery against amebic abscess.

Amebiasis is the third major cause of morbidity and the fourth major cause of mortality from protozoal infection worldwide. Many people infected with *E. histolytica* can be cured without displaying symptomatic disease. However, around 10% of asymptomatic individuals infected with this protozoa develop amebic colitis over the course of a year [4, 5]. Occasionally, the infected individuals develop fulminant amoebic colitis with profuse bloody diarrhea, fever, and abdominal pain [6]. The most common extraintestinal infection of the disease is amebic liver abscess. The trophozoites that breach the colonic mucosa reach the liver through the portal system and induce abscess formation [7, 8]. Thoracic complications of amebic liver abscess are common, as observed in the case described [9]. Sex and age are related to the onset of amebic liver abscess. Among the causes of the high morbidity rates of adult males are alcohol intake and iron storage in the liver. Alcohol intake promotes iron absorption. Chronic alcohol intoxication enhances the deposition of iron in the liver Infants, menstruating females, and the elderly are less prone to amebic liver abscess due to lower liver iron deposition [10].

Intravenous administration of MNZ is the standard therapy for amebic liver abscess [11]. While percutaneous drainage is preferred in pyogenic liver abscesses, there are reports that percutaneous drainage does not have a better prognosis than MNZ in the treatment of uncomplicated cases [12]. However, it is also true that there are amebic liver abscesses resistant to MNZ. Medical treatment depends on MNZ, which has adverse effects including gastrointestinal symptoms and encephalopathy. Resistance to MNZ is an increasing concern [3, 13]. Auranofin, classically used for rheumatoid arthritis, is active against *E. histolytica* and is useful for amebiasis, but its efficacy in humans lacks evidence [14]. Cases where the abscess diameter exceeds 3.0–5.0 cm or where an abscess perforates into other organs are indicated for percutaneous drainage [15–17]. However, reports of surgical drainage have dramatically decreased in recent years. Indeed, a recent report suggested that the mortality rate of cases with surgical drainage was 34%. The lack of preoperative medical treatment with MNZ affected the mortality rate [18]. Surgical drainage in cases with failure of conservative treatment is highly invasive to patients with a poor general condition. The requirement for surgical indication needs to be carefully assessed. Since our patient had amebic liver abscess 15 cm in diameter that was resistant to MNZ and percutaneous drainage, coupled with uncontrolled DIC, we performed an emergency surgery.

Although there are reports of favorable surgical drainage results, only a few descriptions about the surgical methods exist. In our case, by using IOUS in addition to the preoperative CT information, it was possible to determine the liver, its tissue, and abscess cavity. Thus, appropriate drainage was possible. Tan et al. reported that the response rate of surgical drainage to bacterial hepatic abscesses greater than 5 cm was higher than that of percutaneous drainage [19]. They refer to the quality of surgery by confirming the appropriate drainage route with preoperative CT and IOUS, which agrees with our results. However, their cases essentially differed from ours since they performed surgery as an initial treatment for bacterial liver abscess, while our patient underwent secondary treatment of the amebic liver abscess. This involves resection of necrotic tissue with an unclear border and requires more accurate recognition of anatomical structures to avoid damage to major vessels.

Although successful right hepatectomies for amebic liver abscesses have been documented, many patients who are possible candidates for liver abscess operation do not usually have the condition to tolerate extensive hepatectomy. Nevertheless, we did surgical drainage for this case to achieve necrotic tissue removal and place a large-diameter drain into the abscess cavity for effective evacuation that could not be performed by small-diameter percutaneous drainage. Indeed, the inflammatory response was markedly improved after surgery. Although the abscess cavity remained after the operation, eventually the surgically-placed drain worked well to decompress the liver abscess. Determining the timing of surgical drainage for this case was difficult. However, a decision had to be undoubtedly made before the patient could no longer withstand surgery.

Although many modalities like CT or MRI can clearly show liver abscesses, diagnosing whether an abscess is pyogenic or amebic is difficult. *E. histolytica* is rarely detected in patient’s stools or from drained pus. In this patient, we considered that the delay of the diagnosis caused hepatic necrosis refractory to conservative treatment. For patients who have difficulty in receiving treatment for a bacterial liver abscess without proof of amebic existence, it may be possible to complete conservative treatment by administering MNZ before the diagnosis.

In conclusion, MNZ is usually the first choice of treatment for amebic liver abscesses, but surgical drainage under laparotomy using IOUS would be considered a treatment option in cases refractory to conservative management.

## Data Availability

The data supporting the conclusions of this article are included in this published article.

## Abbreviations

MNZ: Metronidazole
IOUS: Intraoperative ultrasonography
CT: Computed tomography
